# Sustained Local Delivery of Diclofenac from Three-Dimensional Ultrafine Fibrous Protein Scaffolds with Ultrahigh Drug Loading Capacity

**DOI:** 10.3390/nano9070918

**Published:** 2019-06-26

**Authors:** S. M. Kamrul Hasan, Ran Li, Yichao Wang, Narendra Reddy, Wanshuang Liu, Yiping Qiu, Qiuran Jiang

**Affiliations:** 1 Key Laboratory of Textile Science &Technology, Ministry of Education, College of Textiles, Donghua University, Shanghai 201620, China; 2 Engineering Research Center of Technical Textiles, College of Textiles, Donghua University, Shanghai 201620, China; 3 Center for Incubation Innovation Research and Consultancy, Jyothy Institute of Technology, Thatguni post Bengaluru 560082, India; 4 Donghua University Center for Civil Aviation Composites, Donghua University, Shanghai 201620, China; 5 College of Textiles and Apparel, Quanzhou Normal University, Fujian 362000, China

**Keywords:** sustained local drug delivery, ultrahigh loading capacity, controlled drug release, three-dimensional ultrafine fibrous scaffold, phase separation

## Abstract

The three-dimensional (3D) ultrafine fibrous scaffolds loaded with functional components can not only provide support to 3D tissue repair, but also deliver the components in-situ with small dosage and low fusion frequency. However, the conventional loading methods possess drawbacks such as low loading capacity or high burst release. In this research, an ultralow concentration phase separation (ULCPS) technique was developed to form 3D ultrafine gelatin fibers and, meanwhile, load an anti-inflammatory drug, diclofenac, with high capacities for the long-term delivery. The developed scaffolds could achieve a maximum drug loading capacity of 12 wt.% and a highest drug loading efficiency of 84% while maintaining their 3D ultrafine fibrous structure with high specific pore volumes from 227.9 to 237.19 cm^3^/mg. The initial release at the first hour could be reduced from 34.7% to 42.2%, and a sustained linear release profile was observed with a rate of about 1% per day in the following 30 days. The diclofenac loaded in and released from the ULCPS scaffolds could keep its therapeutic molecular structure. The cell viability has not been affected by the release of drug when the loading was less than 12 wt.%. The results proved the possibility to develop various 3D ultrafine fibrous scaffolds, which can supply functional components in-situ with a long-term.

## 1. Introduction

Ultrafine fibrous tissue engineering (TE) scaffolds with fibers around 50 to 500 nm can promote tissue repair due to the similarity to the extracellular matrix (ECM) structure [1,2,3,4]. However, the recent development in TE scaffolds requires not only the morphological similarity [5] but also additional functions to improve therapeutic efficacy [6,7,8,9]. In this regard, functional components were incorporated into tissue engineering scaffolds, such as anti-inflammatory drugs [8], antibiotics [10], anti-cancer drugs [11], various proteins [6,12], DNA [13], growth factors [14,15,16] and other therapeutic agents [12]. Without circulation, the functional components can be delivered on-site from scaffolds driving by the concentration gradient and along with the degradation of scaffolds. As a result, the functional components with poor stability in plasma or low in solubility in the aqueous phase can be readily delivered to the target area. The total dosage and infusion frequency required for effective treatments can be substantially reduced [14,17,18,19]. Therefore, the ultrafine fibers loaded with functional components are favored once applied as tissue engineering scaffolds.

Currently, electrospinning (ES) is the most used technique to produce ultrafine fibrous scaffolds from various polymers [20]. The functional components can be loaded via adsorption of the fabricated fibers or blending in spinning solutions. As to the adsorption approach, the components loaded are usually distributed on fiber surface with a limited amount and easy to dissociate resulting in an initial burst release. The method of blending can provide better protection to the loaded components. However, there are several problems associated with this method. Firstly, the maximum loading capacity is restricted by the solubility of functional components and the spinnability of the final solutions. Therefore, the required therapeutic dosage on site can hardly be achieved and the therapeutic efficacy is weakened. Secondly, the loaded components move along with fast evaporating solvents during electrospinning and are fixed in the region near fiber surface. The uneven distribution of the loaded components introduces an obvious burst release. The last weakness lies in the way of fiber deposition. Thirdly, fibers produced by the conventional electrospinning process are accumulated on the fiber collector in a layer-by-layer model. The random orientation of fibers only exists in the two-dimensional (2D) plane parallel to the fiber collector. Thus, in the z-direction, the fibers are packed tightly with small fiber gaps. This structure can hardly mimic the three-dimensionally (3D) randomly oriented fibrous architecture in 3D ECMs. A 3D electrospinning technique has been developed to produce 3D ultrafine fibrous scaffolds with a high specific pore volume [1]. This approach required the use of 50 wt.% surfactant, which had the potential to elicit cytotoxicity, lower mechanical properties of fibers and, more importantly, substantially reduce the drug loading capacity. Besides, the solvent systems for 3D electrospinning were still evaporative, and thus, the distribution of loaded drugs was uneven.

An ultra-low concentration phase separation (ULCPS) technique was developed in our previous work. Ultrafine fibrous scaffolds could be produced with 3D random fiber orientation and large fiber gaps (30–140 μm) allowing for deep cell infiltration [2]. The possible mechanism for fiber formation was that solvent crystals (solvent rich phase) were formed during phase separation, and the solute polymer was excluded by the growing crystals and formed high concentration regions along the edges of the crystals. Since the polymer concentration was ultra-low, the amount of polymer was not sufficient to form connected chips, and hence, fine fibers were produced. The solvent crystals were large enough to segregate these fibers and produce large pores during the lyophilization process [21].

Based on the mechanism of ULCPS, we raised the hypothesis that the incorporated components in the ULCPS scaffolds could be excluded from the solvent crystal together with the polymers during phase separation and evenly mixed in the produced fibers. At ultra-low concentrations, the loading capacity can be adjusted in a large range by varying the weight ratio of the incorporated components and polymers, and high loading capacity can be achieved without the limitations of solubility and spinnability. Physiological inflammation reaction is almost unavoidable during tissue repair and has the risk to provoke therapeutic failure [22,23]. In this research, we tried to introduce an anti-inflammatory drug into tissue engineering scaffolds to fulfill in-situ controlled delivery and suppression of inflammation. Thereby, the total dosage could be reduced and avoided long-term transportation of the drug, which might cause complications. Diclofenac was chosen as the representative anti-inflammatory drug and incorporated in gelatin ULCPS scaffolds. The drug loading and release performances were investigated in comparison with electrospun scaffolds.

## 2. Materials and Methods

### 2.1. Materials

Granular gelatin (~100%) and pharmaceutical grade diclofenac sodium salt (>98%) were purchased from Sinopharm Chemical Reagent Co., Ltd. (Shanghai, China). The fibroblast cells (L929) were bought from ATCC (Manassas, VA, USA). Methanethiosulfonate (MTS) reagent (CellTiter 96^®^ Aqueous One Solution Cell Proliferation Assay) was ordered from Promega Corporation (Madisson, WI, USA). Tetramethylsilane (TMS) and dimethyl sulfoxide-D6 were purchased from Sigma-Aldrich (Milwaukee, WI, USA). Citric acid monohydrate (CA, ≥99.5%), sodium hypophosphite monohydrate (SHP, ~98%), acetic acid (>99%) and all the other chemicals were bought from Shanghai Linfeng Chemical Reagent Co., Ltd. (Shanghai, China).

### 2.2. Fabrication of Gelatin 3D Ultrafine Fibers with and without Drug

Scaffolds were prepared via ULCPS as described in our previous work [2]. Briefly, gelatin aqueous solutions were prepared at an ultralow concentration of 0.01 wt.%. The crosslinker CA and the catalyst SHP were dissolved in the gelatin solution at 15% and 7.5% based on the weight of gelatin. Diclofenac sodium was added in the gelatin solution at designed concentrations (0%, 2%, 5%, 10%, 20%, 50%, 100% based on the weight of gelatin). The solutions were then loaded in aluminum molds (30 mL/mold), phase separated at −80 °C for 2 h, and lyophilized for 24 h. The obtained phase separated (PS) scaffolds were crosslinked at 150 °C for 4 h. The scaffolds produced with different amounts of diclofenac were named as 0%PS to 100%PS, respectively.

### 2.3. Fabrication of Gelatin 2D Ultrafine Fibers with and without Drug

For comparison, 2D ES gelatin scaffolds were also prepared. Gelatin powders were dissolved in acetic acid (90 vol.%) at 14 wt.%. CA and SHP were added at the same ratios as described above. Diclofenac sodium could be only loaded at 0, 2 and 5 wt.% (based on the weight of gelatin), because the drug precipitated when the concentration exceeded 5 wt.%. The solutions were stirred for 24 h, loaded in a 10 mL syringe with a 22 gauge needle, and electrospun at 18 kV with a flow rate of 1 mL/h. The distance between the needle tip and the fiber collector was kept at 18 cm. The fiber mats were then crosslinked at 150 °C for 4 h. The 2D scaffolds were named as 0%ES to 5%ES, respectively.

### 2.4. Morphological Observation

To macro-morphologies of the PS and ES scaffolds with the same weight (22 mg) were recorded by a digital camera (D90, Nikon Inc., Melvile, NY, USA). A scanning electron microscope (SEM, TM3000, Hitachi High-Technologies in Europe, Mannheim, Germany) was used to observe the micro-structures of the scaffolds. Samples were first sputter coated with gold and scanned at different magnifications with an acceleration voltage of 25 kV.

### 2.5. Specific Pore Volume

The specific pore volumes of PS and ES scaffolds were calculated by the following equation:

(1) $$V_{sp}=\frac{V_{p}}{m}=\frac{V_{t}}{m}-\frac{1}{\rho }$$

where $V_{sp}$ is the scaffold’s specific pore volume, m is the dry weight of the scaffold, $V_{p}$ is the total volume of pores in the scaffold, $V_{t}$ is the total volume of the scaffold, and p is the material density. The dry masses and total volumes of scaffolds were measured after heating the scaffolds at 50 °C for 12 h. To obtain the density of gelatin, films were made by casting from the pure gelatin solution (12 wt.%) and the gelatin solutions with diclofenac at different drug ratios. The densities were then calculated based on the volumes of films and their dry weights.

### 2.6. Fourier Transformation Infrared (FTIR) Spectra

FTIR spectra of the original and heated (150 °C for 4 h) diclofenac sodium powders were obtained to confirm the influence of crosslinking procedure on drug configuration. To reveal the interactions between the drug and gelatin during the crosslinking process, the FTIR spectrum of the released substances from the 20%PS scaffold was compared with the spectrum of gelatin powders. The 20%PS scaffolds were first washed in distilled water three times at 0 °C and then soaked in the release bath of distilled water for two days. After removing the scaffolds, the released bath was lyophilized before testing. All the samples were characterized by an FTIR spectrophotometer (Nicolet 6700) (Thermo Scientific, Waltham, MA, USA) at a range of wavenumber from 4000 to 600 cm^−1^ according to the KBr disc method.

### 2.7. NMR Analysis

For ^1^H-NMR and ^13^C-NMR analysis, the powders of diclofenac sodium were pre-heated at 150 °C for 4 h. The solutions for the test were prepared by dissolving the original and treated diclofenac (100 mg) in dimethyl sulfoxide-D6 (1 mL, D, 99.9%), shaking on a vortex mixer for 5 min and then sonicating for 10 min at 400 Hz. The solutions were centrifuged at 4000 rpm for 10 min and the supernatant of each sample (500 μL) was taken for analysis after adding TMS as an internal standard. ^1^H-NMR and ^13^C-NMR spectra of samples were recorded on a Bruker Avance 600 MHz spectrometer at 500 MHz and 125 MHz, respectively.

### 2.8. Zeta Potential

Gelatin powders were dissolved in distilled water at 40 °C with a concentration of 20 wt.%. The gelatin solution was then injected into a pre-cooled water bath (around 0 °C) to form a suspension of gelatin particles. The pHs of the suspensions were adjusted to the designed values using hydrochloric acid (HCl) and sodium hydroxide (NaOH) solutions. The zeta potentials of gelatin particles in distilled water were measured on a Delsa Nano C Particle Analyzer (Beckman Coulter Inc., Brea, CA, USA). The isoelectric point was calculated from the Gauss simulated curve.

### 2.9. Determination of Drug Loaded

Drug-loaded scaffolds were washed in distilled water three times at 0 °C, and then lyophilized and weighted. The scaffolds were hydrolyzed in 2M aqueous NaOH solution at 50 °C for 1 h and diluted with 2M NaOH solution to achieve a gelatin concentration of 0.1 mg/g. The diluted solutions were scanned on a UV/Vis spectrophotometer at the wavelength of 275 nm. The hydrolyzed gelatin solution without the drug in 2M NaOH served as the background. The drug loading efficiency ($E_{LD}$%) and the drug loading capacity ($W_{LD}$%) were calculated by the following equations:

(2) $$E_{LD}\%=E_{LD}\%=\frac{W_{ld}\left(\mathrm{mg}\right)}{W_{d}\left(\mathrm{mg}\right)}\times 100$$

(3) $$W_{LD}\%=\frac{W_{ld}\;\left(\mathrm{mg}\right)}{W_{g}\left(\mathrm{mg}\right)}\times 100$$

where $W_{g}$ is the weight of gelatin, $W_{d}$ is the initial weight of drug for loading, and $W_{ld}$ is the weight of the loaded drug in each scaffold which was calculated based on a pre-established calibration curve.

### 2.10. Drug Release

Samples were washed, lyophilized and weighted as described previously. Before the test, all the samples were sterilized at 120 °C for 1 h. The drug release profile was estimated in the sterilized phosphate buffered solution (PBS, pH 7.4). The scaffolds were immersed in PBS at a fiber to PBS ratio of 1:1000 and incubated at 37 °C with shaking at 40 rpm. At predetermined time intervals, the release medium (1 mL) was taken from each release bath, diluted to 5 g with sterilized PBS and centrifuged at 12,000 rcf for 15 min. The supernatant was scanned on a UV/V is spectrophotometer at the wavelength of 275 nm with PBS as the background. The amounts of drug released were calculated based on a pre-established calibration curve. For each time point, three individual specimens were prepared.

### 2.11. Degradation Evaluation

The weight losses of samples in PBS were measured to perceive the degradation behavior of the drug-loaded scaffolds. The scaffolds were washed, lyophilized, weighted, sterilized, and incubated in PBS as described for the drug release test. At the designed time intervals, scaffolds were harvested from PBS baths, washed three times in distilled water and lyophilized. The dry weight of each sample was measured after lyophilization, and the weight loss percentage was calculated by the equation below:

(4) $$W_{L}\%=\frac{W_{sb}-W_{sa}}{W_{sb}}\times 100$$

where $W_{L}$% is the weight loss percentage, and $W_{sb}$ and $W_{sa}$ are the dry weights of the scaffolds before and after incubation, respectively.

### 2.12. Cell Viability Evaluation

Cell viabilities were evaluated by the MTS cell proliferation assays. The PS scaffolds (five specimens for each sample, 4 mg for each specimen) were sterilized at 120 °C for 1 h and immersed in PBS for washing three times. Then the scaffolds were moved into 48-well plates loaded with DMEM (0.5 mL/well) and immersed for 4 h. The fibroblast cells (L929) were pre-cultured at 37 °C in humidified 5% CO_2_ atmosphere and passaged every three days. The cells were then harvested and seeded onto scaffolds (1 × 10^5^ cells/mL, 1 mL/well). All the samples were cultured at 37 °C with shaking at 100 rpm for 1 h followed by a static culture for two days. Before MTS assay test, the samples were immersed in PBS for washing for 10 min and then moved to the wells loaded with MTS solutions in DMEM (20 vol%, 1 mL/well) for a further culture at 37 °C for 3 h. Then the MTS solution from each specimen (150 µL) was removed to a 96-well plate. The absorbance at 490 nm of each well was measured on a UV/VIS multiplate spectrophotometer (AMR-100, Hangzhou Allsheng Instruments CO., Ltd., Hangzhou, China) with the blank MTS solution as the blank. The optical densities (OD) of each specimen were calculated by subtracting the average blank reading from the direct reading, and the normalized optical densities were calculated by dividing the OD values with the corresponding weights of specimens in order to eliminate the influence from sample weight.

### 2.13. Statistical Analysis

The one-way analysis of variance with Tukey’s pairwise multiple comparison was used to analyze data. With a confidence interval set at 95%, a statistically significant difference was shown when a *p*-value smaller than 0.05. The data significantly different were labeled by different characters on figures. The error bars shown in figures stood for standard deviations.

## 3. Results and Discussion

### 3.1. Morphologies of Scaffolds

**Figure 1:**
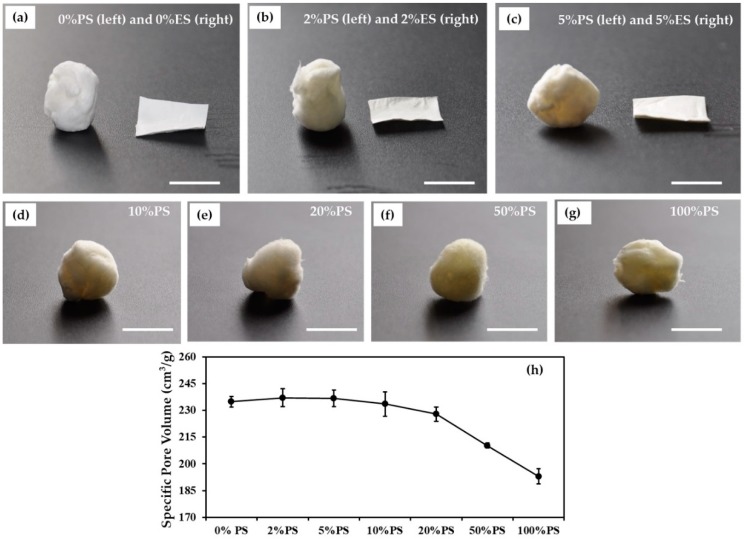
Macro-morphologies of phase separated (PS) and electrospun (ES) fibrous scaffolds with and without diclofenac: (**a**–**c**) digital photos of PS (**left**) and ES (**right**) scaffolds loaded with 0 wt.%, 2 wt.% and 5 wt.% diclofenac, (**d**–**g**) digital photos of PS scaffolds loaded with 10 wt.%, to 100 wt.% diclofenac, (**h**) the specific pore volumes of PS scaffolds. Scale bars indicate 1 cm. (The drug percentage is based on the weight of gelatin).

Figure 1 shows the digital photos of PS and ES scaffolds with the same weight and loaded with different amounts of diclofenac. Compared to the ES scaffolds, the PS fiber bulks occupy much large space (Figure 1a–c). The specific pore volumes of the drug-loaded PS scaffolds are around 237.19 to 193.01 cm^3^/g, while the values of the drug-loaded ES scaffolds are merely about 12.5 cm^3^/g. The macro-morphologies of the PS scaffolds loaded with different amounts of diclofenac are similar (Figure 1d–g), but the specific pore volumes reduced slightly with the increase in the drug amount (Figure 1h).

**Figure 2:**
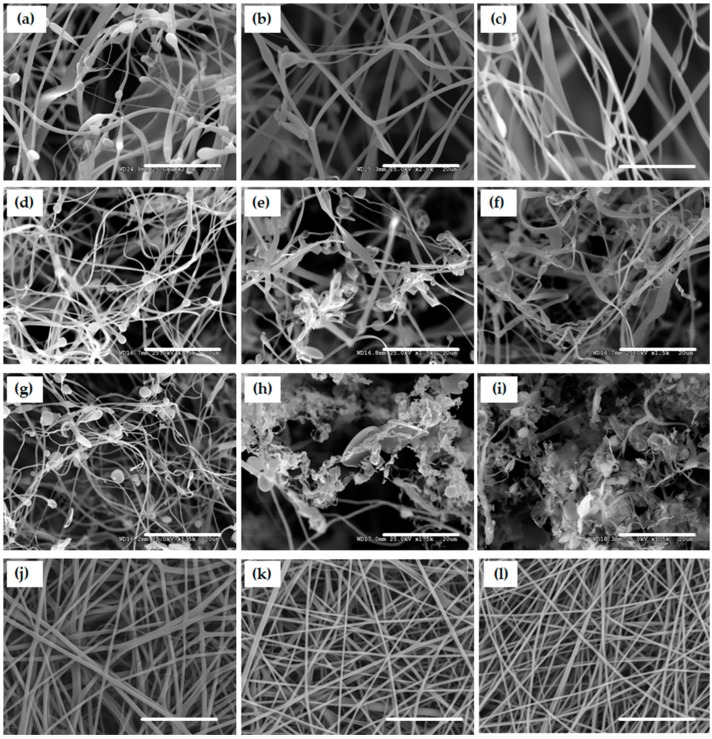
Micro-morphologies of PS and ES scaffolds with and without diclofenac: (**a**–**c**) SEM images of PS scaffolds produced from the gelatin solutions at 0.1 wt.%, 0.05 wt.% and 0.01 wt.%, (**d**–**i**) SEM images of PS scaffolds produced from 0.01 wt.% of gelatin solutions loaded with 2 wt.% to 100 wt.% diclofenac, (**j**–**l**) SEM images of ES scaffolds loaded with 0 wt.%, 2 wt.% and 5 wt.% diclofenac. Scale bars indicate 20 µm. (The drug percentage is based on the weight of gelatin).

Figure 2 displays the micro-morphologies of scaffolds. Fibrous scaffolds could be formed when the concentrations of gelatin solutions varied from 0.1 to 0.01 wt.%. Ultrafine fibers and small beads were observed in the PS sample produced from the 0.1 wt.% gelatin solution. As the concentration decreased, the number of beads and fiber diameter were reduced (Figure 2a–c). Thus, gelatin concentration was set at 0.01 wt.% in the following works. The fibrous structure of scaffolds was maintained when the amount of diclofenac increased from 2 to 20 wt.%. Chips and beads could be seen among fibers (Figure 2d–g). When the amount of drug was raised to 50 and 100 wt.%, the predominant structures were chips and beads (Figure 2h,i).

The mechanism to control the scaffold morphology is still unclear, but assumptions have been made based on previous research. The “solvent crystal templating theory” and the “nucleation and growth theory” might act simultaneously to control the scaffold structure [24,25]. In the solution with a low concentration of solutes, the “solvent crystal templating theory” might play the key role. This theory indicates that the solvent in a solution system forms crystal when the solution temperature decreases below the melting temperature, and the solutes with very limited solubilities in solvent crystals are excluded to the edges of crystals. A concentration gradient of solutes is established in front of crystals and reduces the melting point leading to the formation of a supercooling region. The structure of the crystal template is determined by the destabilizing solute interfacial concentration gradient and the surface energy against crystal growth. The final scaffold morphology is controlled by the crystal template structure [24]. The “nucleation and growth theory” usually occurs in the solutions with high concentrations and at a slow quenching rate. As the solubility of solutes is reduced, small nuclei of solutes are formed and tend to grow into larger crystals [25]. Since the gelatin and drug concentrations used in this research were ultralow, the solution system underwent solvent crystal templating process and formed large ice crystals which created big pores in the final scaffolds. With a limited amount of gelatin, it was difficult to connect the polymer-rich phase into pieces of films and to produce sponges. Consequently, the gelatin molecules were pushed to form fibers along the ice crystal edges (Figure 2c). In the supercooling region, the excluded solutes accumulate and might achieve the critical concentration to trigger nucleation. Therefore, the nucleation and growth process may also act as the key factor in this region. Hence, the solute molecules not only gathered around the crystal edges, but also formed cores in the supercooling region, and grew to beads or chips. It might be the reason that a few beads could be observed in the scaffolds when the gelatin concentration increased from 0.01 to 0.1 wt.%. Diclofenac has a lower solubility than gelatin in water. In the gelatin/diclofenac aqueous solution system, the drug concentration in the supercool region was raised during quenching, and the drug molecules might undergo nucleation first to form cores. Since diclofenac is a salt, it was able to increase the solution viscosity and reduced the thermal conductivity in the supercooling region when the diclofenac amount was high [26]. Therefore, the solvent crystallization process was retarded and triggered the nucleation and growth process of gelatin molecules more readily. As a result, more beads and chips were produced in the 50 and 100%PS scaffolds (Figure 2d–i). Besides, with the increased amount of diclofenac, the repulsion force between negatively charged gelatin and diclofenac was enhanced. This increased repulsion could lower the surface energy against gelatin crystal growth and easy to form big beads and chips [25,26].

Contrarily, the addition of diclofenac in the ES spinning solutions did not substantially change the structures of the ES scaffolds (Figure 2j–l), but merely reduced the fiber mean diameters from 1041 nm (0%ES) to 666 nm (2%ES) and 647 nm (5%ES). The conductivities of the spinning solutions might be elevated by diclofenac, resulting in a stronger extrusion to produce finer fibers [27].

### 3.2. Drug Loading Performance

**Figure 3:**
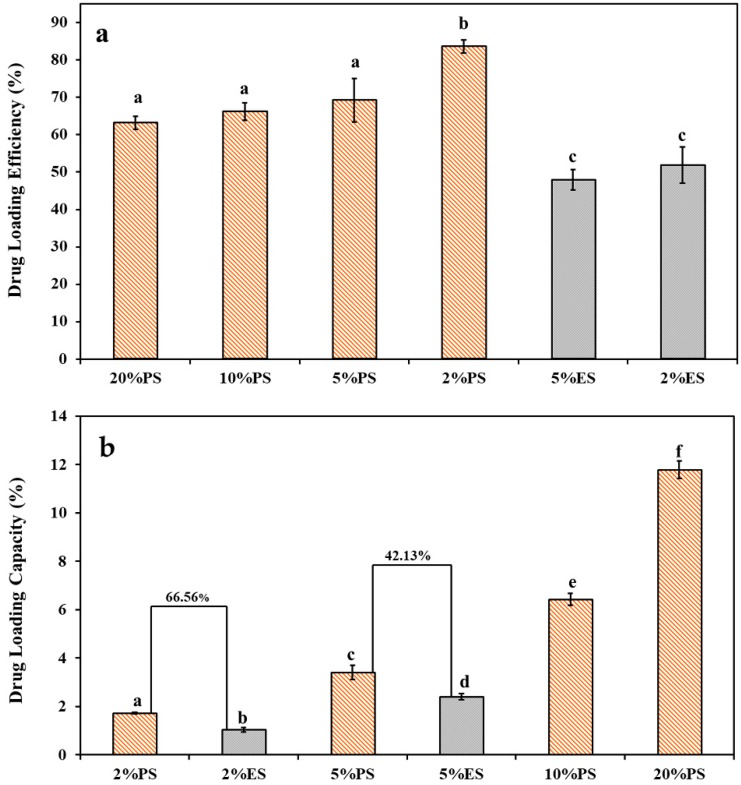
Comparison of drug loading behaviors between the drug-loaded PS and ES scaffolds: (**a**) drug loading efficiencies, (**b**) drug loading capacities. Significant differences among data were labeled by different characters.

The drug loading properties are shown in Figure 3a. The loading efficiencies (calculated via Equation (2)) of all PS scaffolds were above 60%. When the initial drug concentration was the lowest (2 wt.%), the highest loading efficiency (83.56%) could be achieved. As the initial drug concentration increased from 2 to 5 wt.%, the drug loading efficiency was reduced to 69.19%, but a further elevation in the initial drug concentration up to 20 wt.% displayed no significant effect on the drug loading efficiency. Compared to the scaffolds produced by electrospinning (2%ES and 5%ES), the corresponding PS samples (2%PS and 5%PS) showed 61.2% and 44.4% elevations in the drug loading efficiencies, and 66.5% and 42.1% increments in the drug loading capacities (calculated via Equation (3)) (Figure 3b).

**Figure 4:**
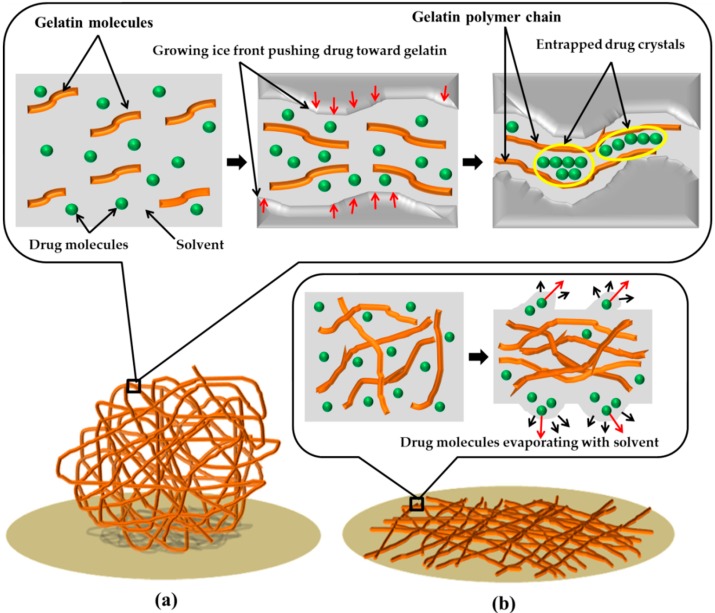
Scheme of the drug loading mechanisms in PS and ES scaffolds: (**a**) scheme for PS scaffolds, (**b**) scheme for ES scaffolds.

These results indicated that the phase separation approach was more efficient to encapsulate drug molecules with a much higher capacity. The reasons might lie in the different mechanisms of the two fiber formation processes. As shown in Figure 4a, the loading of diclofenac into gelatin scaffolds was driven by the growing and exclusion of the ice crystals. Since the solution can be totally frozen, the maximum drug loading capacity is determined by the maximum drug/gelatin ratio at which the integrity of final scaffolds can still be maintained. On the contrary, in the electrospinning system, the upper limit of the drug loading amount must be lower than the drug saturation concentration in the spinning solution and, meanwhile, allow for a stable spinning. Therefore, the maximum capacity of drug loaded in ES scaffolds is restricted and substantially lower than that in the PS scaffolds. The phase separation process took around 1 h and allowed for well embedding of diclofenac in the PS scaffolds (Figure 4a). In contrast, the electrospinning process was accomplished in less than a second. The rapid evaporation of solvent drove drug molecules towards the surface of the solution and resulted in a higher concentration of drugs close to the fiber surfaces (Figure 4b). Hence, the drugs were more ready to be removed during the washing and release processes.

### 3.3. Drug Release Profile

**Figure 5:**
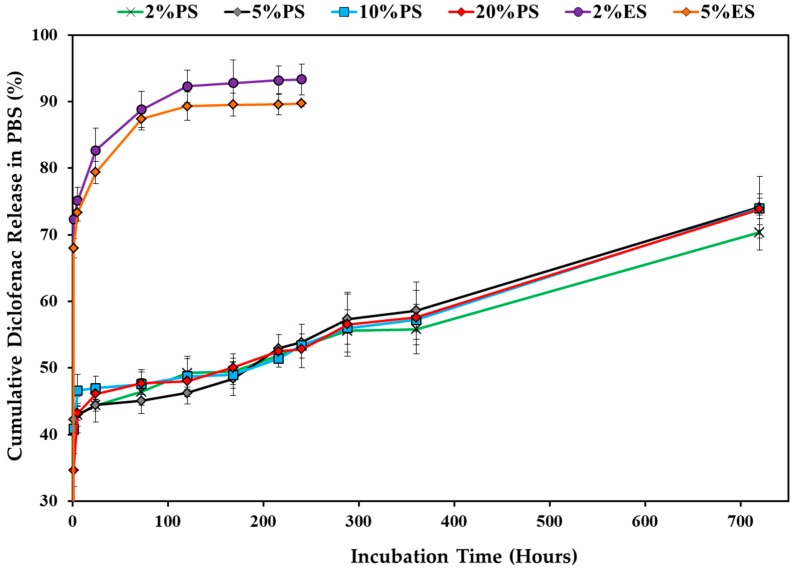
Drug release profiles of the PS and ES scaffolds in phosphate buffered solution (PBS) at 37 °C for up to 30 days.

Figure 5 provides the drug release profiles of the drug-loaded ES and PS scaffolds. The 2%ES and 5%ES samples showed obvious burst releases within 1 h (72.3% and 69.9%). After one week, 92.8% and 89.5% of diclofenac had been released from these ES scaffolds, and then the release profiles leveled off. On the contrary, the initial release of the PS scaffolds (1 h) was substantially reduced (34.7% to 42.2%), even when the drug loading amounts were times higher. In the following 30 days, the release profiles of different PS scaffolds were similar and displayed a linear trend with a release rate of around 1% per day. At day 30th, around 70.4% to 74.2% of diclofenac was released, and the release profile had not leveled off. It could be deduced that the daily dosages of drugs provided by the PS scaffolds were able to be well controlled by adjusting the initial drug concentrations in the phase separation solutions.

**Figure 6:**
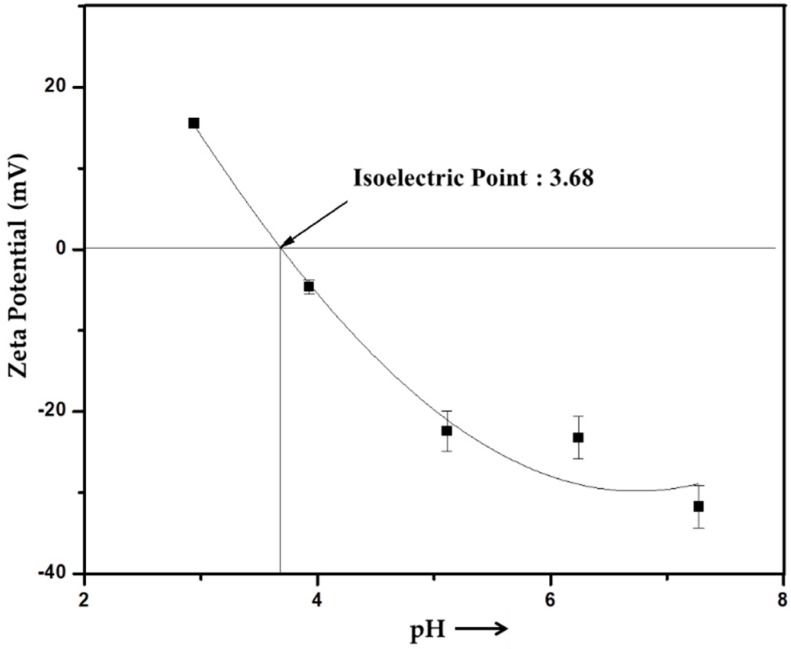
Zeta potential of gelatin in water.

Since the ES and PS scaffolds displayed different initial drug release patterns, it partially proved the hypothesis about the differences in the drug distributions described in Figure 4. In the ES fibers, a large amount of drug was located near the surfaces and was easy to release, while the drugs were encapsulated in the PS fibers more evenly. When the drugs at the top layers were released, those in the center were slowly diffused out and released along with degradation. Thus, the PS scaffolds showed substantially reduced burst releases. For real applications, better strategies can be used, such as providing a surface protection layer or a pre-washing step. The repulsion between the diclofenac molecules and gelatin molecules might serve as one of the reasons for the burst release profiles of both PS and ES scaffolds. With an isoelectric point at 3.68 (Figure 6), gelatin carries strong negative charges at pH 7.4. While diclofenac molecules also bear negative charges. Thus, the repulsion forces between them promoted the initial drug release closed to the fiber surface. It can be deduced that the initial drug release would be weakened if either the carrier polymer or the selected drug was replaced by a positively charged one.

### 3.4. Degradation Behavior of the Drug Loaded Scaffolds

**Figure 7:**
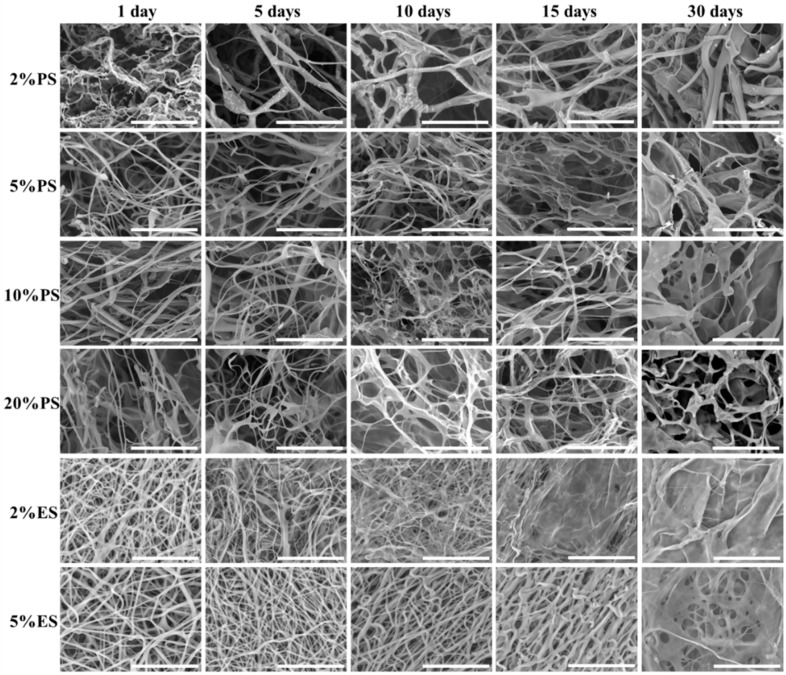
Morphological changes of drug loaded PS and ES scaffolds after incubation in PBS at 37 °C for up to 30 days. Scale bars indicate 30 µm.

The changes in scaffold morphology were displayed in Figure 7. Once immersed in PBS, all gelatin scaffolds swelled, but the fibrous structure could be kept. From day 5, fibers started to fuse and form thicker ones. Thin films were built among fibers. These phenomena became more obvious along with incubation. After 30 days, films were the major structure in scaffolds. The PS scaffolds showed porous structures after 30 days, while the ES scaffolds became more compact and the structure with interconnected pores could hardly be maintained.

**Figure 8:**
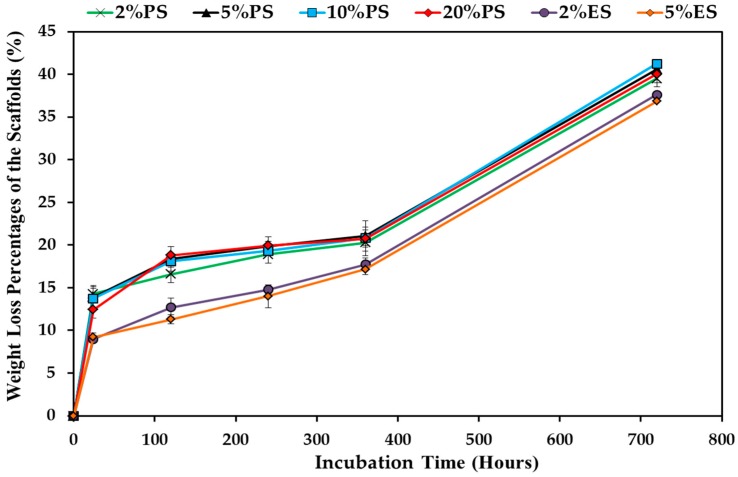
Weight loss percentages of drug loaded PS and ES scaffolds in PBS for 30 days.

The degradation behaviors of scaffolds have also been described as weight losses (calculated via Equation (4)) shown in Figure 8. After the first day, the weight losses of all PS scaffolds were around 13 to 14 wt.%. The ratios were almost 50% higher than the weight loss ratios of the ES scaffolds (9.1 wt.%). The possible reason might be that the fibers in the ES scaffolds were close to each other, and the released small molecules, such as diclofenac, short protein chains, and unfixed CA and SHP, might form a high concentration region around fibers, and hinder the further release and degradation of these components from fibers. On the contrary, the loose structures of the PS scaffolds allowed a quick removal of the released components and promoted the initial weight losses. Although the absolute drug release amounts of the ES scaffolds were lower than the values of the PS scaffolds (ES: 5.7~9.4 mg/g of fiber), the drug release percentages of the ES scaffolds were much higher than the release percentages of the PS scaffolds as shown in Figure 5, because the initial drug loading amounts of PS scaffolds were around 1.4 to 1.6 folds of the amounts in ES scaffolds. In the following 30 days, the weight losses of the PS scaffolds were similar but still higher than the ES scaffolds, whereas the weight loss profiles of all scaffolds were almost similar with roughly three stages. From day 1 to day 15, the 2%ES and 2%PS scaffolds showed a linear weight loss profile, while other samples displayed a quicker weight loss rate before day 5 and a slower weight loss rate in the flowing 10 days. In this stage, the average weight loss rates of samples were around 0.25% per day. From day 15 to day 30, the weight loss rates of all samples increased to 1.29% per day. The degradation of gelatin might be the major factor to control the weight loss speed. A similar phenomenon was observed in our previous work [2]. The quick weight losses of the crosslinked gelatin ultrafine fibrous scaffolds started from day 15.

### 3.5. Chemical Structure Confirmation

**Figure 9:**
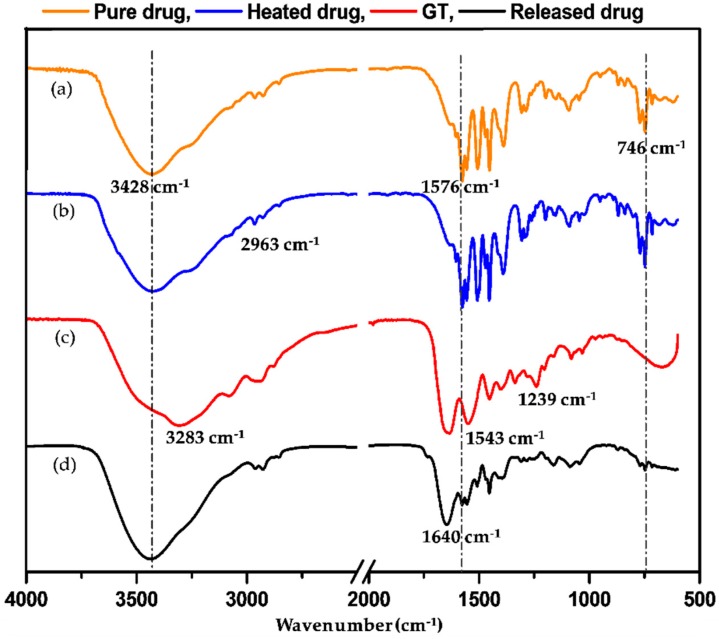
FT-IR spectra: (**a**) untreated diclofenac sodium, (**b**) heated (150 °C for 4 h) diclofenac sodium, (**c**) gelatin, (**d**) released diclofenac sodium.

The FT-IR spectra of the original drug, the heated drug (under crosslinking conditions), the released drug and gelatin powders are shown in Figure 9. The spectrum of the original diclofenac shows the characteristic peaks at 3428 cm^−1^, 2963 cm^−1^, 1576 cm^−1^, and 746 cm^−1^ (Figure 9a), corresponding to the –NH– stretch vibration of the secondary amine groups, the –C=C– stretch vibration of the phenyl structures, the –CH– stretch of the aromatic structure and the stretch of the phenyl group substituted by chloride [28,29]. The chloride substituted phenyl group is responsible for the inhibition of cyclooxygenase, which relates to inflammation and pain [30,31]. After heating under the same conditions for crosslinking, no change on the spectrum was observed (Figure 9b), indicating that the drug can withstand the crosslinking temperature. In the spectrum of gelatin (Figure 9**c**), the peak at 3283 cm^−1^ stands for amide-A and free water. The characteristic peaks at 1630 cm^−1^, 1543 cm^−1^ and 1239 cm^−1^ are related to amide-I (the –C=O stretch/hydrogen bonding couple with –COO–), amide-II (the bending vibration of –NH– groups and stretching vibration of –C–N– groups), and amide-III (the in-plane vibrations of –NH– and –C–N– groups). The last spectrum belongs to the released drug harvested from the drug release bath (Figure 9d). A small portion of gelatin was also dissolved from fibers and released in the bath. Therefore, this sample is mainly diclofenac but mixed with gelatin. The corresponding spectrum shows the most characteristic peaks of diclofenac and gelatin, but some peaks shifted. For example, the peaks at 3428 cm^−1^ of diclofenac moved to 3440 cm^−1^, the peaks at 1630 cm^−1^ of gelatin shifted to 1640 cm^−1^, and the peaks at 3283 cm^−1^, 1543 cm^−1^ and 1239 cm^−1^ of gelatin were not observed. These results proved that hydrogen bonds could be built between diclofenac and gelatin. The peak at 746 cm^−1^ and 1576 cm^−1^ of diclofenac was retained, indicating that the functional structure of diclofenac was maintained.

**Figure 10:**
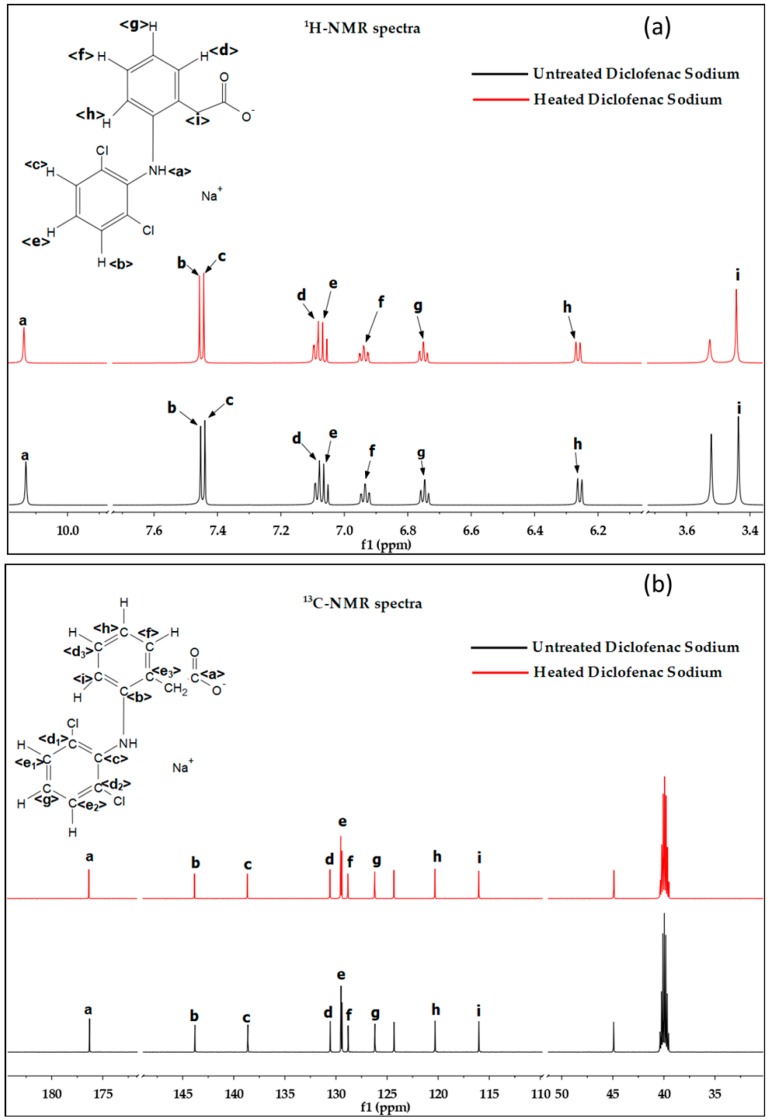
NMR spectra: (**a**) ^1^H-NMR spectra of the raw and heated (150 °C for 4 h) diclofenac sodium, (**b**) ^13^C-NMR spectra of the raw and heated (150 °C for 4 h) diclofenac sodium.

The ^1^H-NMR spectra of the raw and heated diclofenac sodium under the crosslinking conditions are shown in Figure 10a. The characteristic signals were detected in both samples. In the spectra of the original drug, the signal for the secondary amine proton locates at 10.15 ppm (<a>). The signals of 2,6-diclorophenyl ring protons appear in the down field at 7.45 ppm (<b>), 7.44ppm (<c>) and 7.08 ppm (<d>). The phenylacetate ring protons resonate at 7.06 ppm (<e>), 6.93 ppm (<f>), 6.70 ppm (<g>) and 6.25 ppm (<h>). The signal of the methylene proton near the carboxylic group is at an upper location, 3.43 ppm (<i>). The locations and the broadness of the signals were not changed after heating treatment. Similar results are observed in the spectra of ^13^C-NMR (Figure 10b). The signal of carboxylic group carbon locates at 176.28 ppm (<a>). The 2,6-diclorophenyl ring carbons resonate at 138,16 ppm (<c>), 130.60 ppm (<d_1_ and d_2_>), 129.56 ppm (<e_1_ and e_2_>) and 126.19 ppm (<g>), while the phenylacetate ring carbons show signals at 143.80 ppm (<b>), 130.60 ppm (<d_3_>), 129.56 ppm (<e_3_>), 128.82 ppm (<f>), 120.34 ppm (<h>) and 116.02 ppm (<i>). The results of the ^1^H-NMR and ^13^C-NMR spectra indicate that the structure of diclofenac sodium was preserved under the crosslinking conditions.

### 3.6. Cell Viability

**Figure 11:**
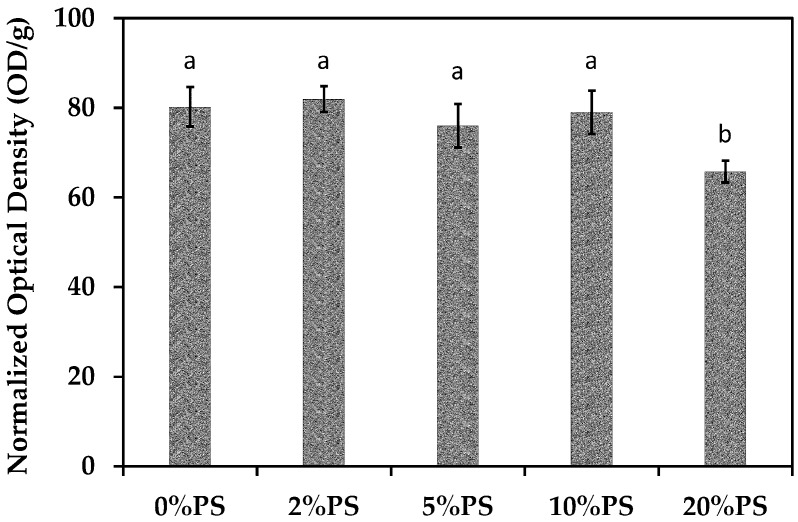
Cell viabilities after culture for two days on PS scaffolds evaluated by MTS assay. Significant differences among data were labeled by different characters.

Figure 11 shows cell viability evaluation results by MTS assay. After culture for two days, the cell viabilities on the pure gelatin PS scaffold and the PS scaffolds loaded with diclofenac less than 20 wt.% were similar. As the drug concentration achieved 20 wt.%, the cell viability displayed a 17% reduction. Although all the PS scaffolds have shown similar release profiles, the absolute amount of drug released from the 20%PS sample must be higher than the rest. After culture for two days, the accumulated drug concentration might achieve a critical value to induce suppression of cell growth. However, for real applications, the released drug might accumulate due to the drug uptaking and circulation around the tissue repairing site.

## 4. Conclusions

In this research, a one-step ULCPS process was developed to produce 3D ultrafine fibrous scaffolds loaded with a high amount of the functional component, diclofenac. The maximum drug loading capacity and drug loading efficiency were about 12 wt.% and 83.56%, which were around 391.74% and 61.23% higher than the maximum values of the ES scaffolds. The drug structure could be maintained during fabrication. Although with an ultrahigh drug loading amount, the PS scaffolds displayed a 40% reduction in the initial release percentages compared to the ratio of the ES scaffolds. Sustainable linear release profiles were observed in the following 30 days, around 1% per day. Besides, the PS fibrous scaffolds possessed ultrahigh specific pore volumes around 237.12 to 227.9 cm^3^/mg and could maintain the fibrous structure with incubation for 30 days. The incorporation of the drug did not affect cell growth when the drug loading was less than 12 wt.%. This research provided a possible approach to overcome the loading limitation by solubility or spinnability and produce 3D ultrafine fibers with high loading capacities and specific pore volumes. These scaffolds could not only support 3D tissue repair but also offer sustained in-situ delivery of functional components.
